# Response of Fungal Communities and Co-occurrence Network Patterns to Compost Amendment in Black Soil of Northeast China

**DOI:** 10.3389/fmicb.2019.01562

**Published:** 2019-07-09

**Authors:** Wei Yang, Xuyuan Jing, Yupeng Guan, Cheng Zhai, Tao Wang, Dengyu Shi, Wenpeng Sun, Siyu Gu

**Affiliations:** ^1^College of Resources and Environment, Northeast Agricultural University, Harbin, China; ^2^Institute of Crop Research, Heilongjiang Academy of Land Reclamation Sciences, Jiamusi, China; ^3^Institute of New Rural Development, Northeast Agricultural University, Harbin, China

**Keywords:** compost addition, growth stage, soybean, fungi, Miseq sequencing, network analysis

## Abstract

In agroecosystems, fungi not only attract attention as crop pathogens, but also play crucial roles in nutrient cycling as decomposers and arbuscular mycorrhizal mutualists. Consequently soil fungi strongly influence agroecosystem function, and are conspicuously influenced by agricultural practices. We examined the effects of four compost rates (0, 11.25, 22.5, and 45 Mg ha^−1^) on soil fungal community compositions and network patterns in soybean at seedling, flowering, and mature stage in a field experiment in black soil of Northeast China. Miseq sequencing was used to characterize the soil fungal community. Our results revealed that soil fungal richness was unaffected by compost addition, while soil fungal community composition was significantly influenced by compost addition across the growing season. Among the combined “top 20” fungal OTUs, 15 OTUs positively responded to compost addition, while 10 negatively responded. The abundance of predicted pathotroph was greatly decreased by the 45 Mg ha^−1^ compost addition. Network analysis indicated that the fungal networks in compost amended soils were more complex and harbored more positive links than the control. Fungal network harbored more positive links among saprotroph-saprotroph and saprotroph-symbiotroph in moderate level of compost amended soils than other networks. In conclusion, this study revealed that compost addition impacted positively both the soil fungal communities and network patterns within a single growing season. Thus, compost addition could be a good practice to enhance the soil fungal community and function and ultimately soil health and quality.

## Introduction

Black soil is one of the most fertile soils for crop production, and is widely distributed in Northeast China (Xu et al., 2010; Yin et al., 2015). However, owing to the increasing population and demand for grains, excessive chemical fertilizers have been applied in this region for decades, and this has resulted in environmental pollution, deterioration in soil quality, and loss of biodiversity (Liu et al., 2010). To mitigate these problems, organic farming systems have gained increasing importance in this region (Ding et al., 2017; Hu et al., 2017). Organic amendments are used primarily to enhance plant nutrition and crop yield (Ding et al., 2017), but they can also alter soil microbial composition and diversity (Guo et al., 2018) because the substrates they supply can be utilized by the microorganisms (Sun et al., 2016). Fungi are one of the most abundant soil inhabiting microbes (Finlay, 2002). In agroecosystems, fungi serve as important decomposers and play a significant role in organic turnover. They also form mutualistic symbiotic associations with plant roots to improve nutrient uptake (Smith and Read, 2008). Consequently, soil fungi strongly influence agroecosystem functioning (Hijri, 2016).

Application of inorganic fertilizers generally decrease soil pH and increase soil compaction (Guo et al., 2010), which have previously been reported to negatively affect soil fungal abundance and diversity (Lanzén et al., 2015). Alternatively, organic fertilizers supply substrates for saprotrophic fungal growth and provide a sustained supply of nutrients without a negative impact on soil pH (Yang et al., 2017; Zhang J. et al., 2018). Organic amendment generally promotes higher abundance and diversity of soil fungi (Song et al., 2015; Hu et al., 2017), and shifts in soil fungal community composition in response to organic amendment have been documented in various agroecosystems (Hartmann et al., 2015; Sun et al., 2016; Xue et al., 2018). However, most of these studies only reported on short-term or long-term effects in a single sampling time (Sun et al., 2016; Hu et al., 2017; Xue et al., 2018), while only a few researches focused on a course of time (Zhen et al., 2014; Yang et al., 2018). Furthermore, it was previously proposed that soil microbial communities would be either resilient to disruption and quickly recover to its initial state or resistant to disturbance and may not change (Allison and Martiny, 2008). Therefore, these studies only capture a specific status of soil fungi that may not represent their true response. For this reason, a time course study is needed to reveal the dynamics of fungal communities in response to organic amendment.

Soil fungi vary in their substrate preferences, and mechanisms for nutrient acquisitions, therefore fungi exhibit a range of trophic status and are functionally distinct (Dickie, 2007). Thus, fungi could form complex interactions (predation, parasitism, competition, amensalism, commensalism, and mutualism) with each other, and these interactions determine the overall soil fungal community structure and ecosystem stability (Olesen et al., 2007; Deng et al., 2012). Network analysis has proven to be a powerful tool to explore the complex interactions that exist among microbes, and it’s a possible approach to derive meaningful information beyond community analyses (Banerjee et al., 2016). Although, enhanced complexity in soil bacteria co-occurrence network as a result of organic input application has been previously reported (Ling et al., 2016; Zhou et al., 2019), the response of fungal co-occurrence network pattern to the organic amendment is less understood.

To address the aforementioned knowledge gaps, a compost addition experimental system was established in an agroecosystem on the Songnen Plain in black soil to (1) determine the dynamics of soil fungal communities in response to compost addition during the growing season; (2) determine whether the compost addition would influence soil fungal co-occurrence network pattern; (3) explore the interactions among different trophic status in response to compost addition. We hypothesized that compost application would enhance soil fungal richness, and change the fungal community compositions and co-occurrence network patterns.

## Materials and Methods

### Field Experiment Design

The field experiment was carried out from May to September 2016 at the Xiangyang experimental farm of Northeast Agricultural University, Harbin, China (45°45′ N, 126°54′ E). The field has been in maize-soybean crop rotation, with chemical fertilizers applied, before 2016. The soil at this study site is a typical black soil (classified as Mollisols, according to USDA soil taxonomy). Soybean [*Glycine max* (L.) Merrill], which is a staple crop in Northeast China, was used as the test crop. Compost were applied as basal fertilizer and evenly mixed with top soil before soybean was planted. The compost was obtained through an on-farm composting of cow manure and maize straw in Shuangcheng City; details of the compost properties can be found in Yang et al. (2017). Compost addition rates of 0, 11.25, 22.5, and 45 Mg ha^−1^ were applied in this study. These treatments are referred as control (CK), low level of compost addition (LC), moderate level of compost addition (MC), and high level of compost addition (HC). The compost addition rate in LC treatment was approximately equal to 200 kg N/ha (equivalent to the recommended amount of N fertilizer in this area). Each treatment covered an area of 4.5 m × 5 m with four replicates, which resulted in 4 × 4 (16) plots in total. Plots were randomly distributed with 2 m in between plots (inter and intra row spacing). The soybean cultivation systems were all rotary plowed, ridged, and ditched to 5 cm depth before planting. The compost and seeds were spread into the ditch, and then mixed with soil homogeneously. Weeds were removed manually twice during the growing season, and no herbicide, pesticide, or fungicide were added. For climate and soil characteristics see Yang et al. (2017).

### Sampling and Soil Variables

Soil sampling was conducted on June 4 (seedling stage), July 24 (flowering stage), and August 27 (mature stage) in 2016. In each plot, five soil cores (0–20 cm deep, 5 cm diameter) were randomly collected and homogenized to form one single sample per plot for each sampling time. The freshly collected soil samples from each plot were divided into two parts. The first part was sieved (<1 mm) and stored at −80°C for DNA extraction, and the remaining part was air dried and processed for estimation of the physicochemical parameters. Soil organic matter (SOM), total phosphorus (TP), total nitrogen (TN), available phosphorus (AP), available potassium (AK), available nitrogen (AN), pH and electrical conductivity (EC), and soil moisture (SM) were determined and presented in Yang et al. (2017).

### Miseq Sequencing

For each sample, total genomic DNA was extracted from 0.25 g of frozen soil with the PowerSoil DNA Isolation Kit (MoBio Laboratories, Inc., Carlsbad, CA, United States) following the manufacturer’s protocol. An approximately 300–350 bp region of the Internal Transcribed Spacer 2 (ITS2) region was amplified with forward primer gITS7 (Ihrmark et al., 2012) and reverse primer ITS4 (Gardes and Bruns, 1993). Primer gITS7 contained a unique 12 nt barcode at the 5′ end for Miseq sequencing detection. Miseq sequencing was performed using 2 × 250 pair-end method. The raw sequence data had been accessioned in the Sequence Read Archive of National Center for Biotechnology Information, United States (SRA accession: SRP151204). More details about the PCR conditions and quality assessment are provided in Supplementary Table S1.

### Bioinformatic Analysis

Raw sequences shorter than 250 bp, with ambiguous base “N,” and average base quality score <20 were removed using QIIME Pipeline Version 1.8.0 (Caporaso et al., 2010). The ITS2 region was extracted using ITSx (Bengtsson-Palme et al., 2013) and potential chimeric sequences were deleted using the “chimera.uchime” command in Mothur (Schloss et al., 2009) with UNITE (Nilsson et al., 2015) reference database. The remaining sequences were clustered into different operational taxonomic units (OTUs) with 97% similarity level using the UPARSE pipeline (Edgar, 2013). The representative sequences of each fungal OTU were blasted against the UNITE database to assign taxonomic annotation. Plant and protozoa OTUs were then removed from the dataset. The number of sequences per sample was rarefied to the same sample size using the “sub.sample” command in the Mothur (Schloss et al., 2009). Accumulative numbers of fungal OTUs were calculated using the “rarefy” function in the package vegan (Oksanen et al., 2013) in R (R Core Team, 2013).

### Statistical Analysis

Soil fungal OTU richness, Shannon diversity, Simpson, and Pielou evenness indices were calculated in the vegan package (Oksanen et al., 2013) in R (v.3.1.1) (R Core Team, 2013). Significant effects of compost addition on fungal OTU richness, Shannon diversity, Pielou evenness indices and the relative abundance of abundant fungal phylum were examined by one-way ANOVA in seedling, flowering, and mature stage. All data meet the assumption of normality and homogeneity of variance. Differences among treatments were then tested by a Tukey’s HSD *post hoc* test (*P* < 0.05). The relative abundance of some abundant fungal OTUs did not meet the normal distribution, therefore the Kruskal–Wallis test was conducted to examine the effect of compost addition on the abundant OTUs in seedling, flowering, and mature stage. Fungal OTUs that could be assigned to genus were extracted, and indicator species analysis was conducted to determine genera that were significantly associated with each treatment (genus with Indval values >0.3 and *P* < 0.05 are strong indicators) using the function “indval” in the labdsv package (Roberts, 2010).

The significant effects of compost addition, growth stage, and their interaction on soil fungal community composition were examined using permutational multivariate analysis of variance (PERMANOVA) in the vegan package (Oksanen et al., 2013). Subsequently, the fungal community composition was ordinated using non-metric multidimensional scaling (NMDS) with the dissimilarity matrices using the “metaMDS” function in the vegan package (Oksanen et al., 2013). Mantel tests were applied to explore the correlations between soil fungal communities and soil variables (SOM, TN, TP, AN, AP, AK, and pH) in the ecodist package (Goslee and Urban, 2007). The heatmap of combined “top 20” fungal OTUs was generated in the pheatmap package to evaluate the variations of abundant OTUs among treatments. The analyses above were carried out in R (v.3.1.1) (R Core Team, 2013). The fungal functional groups (pathotroph, saprotroph, symbiotroph) were characterized using FUNGuild v1.0 (Nguyen et al., 2016).

The Random Matrix Theory (RMT) based network analysis was performed using the Molecular Ecological Network Analyses Pipeline (Deng et al., 2012). Four co-occurrence networks of soil fungi from CK, LC, MC, and HC treatments were built using data from all three sampling times. After the network construction, the same thresholds were determined in all networks. The “global network properties,” the “individual nodes’ centrality,” the “module separation and modularity,” and the “randomize the network structure and then calculate network properties” were calculated in the pipeline. Gephi (Bastian et al., 2009) was used to visualize the network. The topological roles of each node were evaluated by the threshold values of Zi and Pi as proposed by Guimera and Amaral (2005).

## Results

### Sequencing Data Analysis and Fungal Diversity

A total of 1,314,019 fungal reads were obtained after quality control, from which 108,088 potential chimeras were removed. The remaining 888,201 non-chimeric reads were assigned to 1,882 operational taxonomic units (OTUs) based on a 97% sequence similarity. Of these 1,882 OTUs, 1042 (905,802 reads) belonged to fungi. As the number of fungal reads ranged from 8,296 to 44,764 among samples, the read numbers were normalized to 8,296, resulting in a normalized dataset containing 1,018 fungal OTUs (398,208 reads).

The fungal OTU richness, Pielou evenness, Shannon diversity, and Simpson indices among treatments ranged from 218 to 292, from 0.6 to 0.67, from 3.26 to 3.74, and from 0.87 to 0.93, respectively (Table 1). However, the effects of compost addition on these alpha-diversity indices were not significantly different (*P* < 0.05) across the growing season (Supplementary Table S2). Although, a decreasing trend of fungal OTU richness, Pielou evenness, Shannon diversity, and Simpson indices along with the compost rate was observed in seedling stage (Table 1).

**Table 1:** Soil fungal richness, Shannon diversity, Simpson diversity, and Pielou evenness indices among treatments in seedling, flowering, and mature stage.

		Richness	Shannon	Simpson	Pielou
Seedling	CK	265 ± 30.77	3.74 ± 0.17	0.92 ± 0.03	0.67 ± 0.03
	LC	261 ± 13.54	3.58 ± 0.16	0.93 ± 0.01	0.64 ± 0.03
	MC	232.5 ± 36.97	3.47 ± 0.45	0.92 ± 0.05	0.64 ± 0.06
	HC	218 ± 28.60	3.26 ± 0.08	0.90 ± 0.01	0.61 ± 0.01
Flowering	CK	246.5 ± 20.86	3.49 ± 0.21	0.92 ± 0.03	0.63 ± 0.03
	LC	256.25 ± 23.96	3.48 ± 0.33	0.91 ± 0.03	0.63 ± 0.05
	MC	266 ± 37.57	3.51 ± 0.20	0.92 ± 0.03	0.63 ± 0.03
	HC	263.25 ± 7.46	3.58 ± 0.04	0.93 ± 0.01	0.64 ± 0.01
Mature	CK	245.25 ± 50.69	3.28 ± 0.63	0.87 ± 0.09	0.60 ± 0.09
	LC	292.25 ± 9.74	3.63 ± 0.28	0.92 ± 0.03	0.64 ± 0.05
	MC	285.75 ± 21.65	3.66 ± 0.20	0.92 ± 0.02	0.65 ± 0.03
	HC	272.25 ± 34.56	3.67 ± 0.30	0.93 ± 0.02	0.66 ± 0.04

*CK, control; LC, low level of compost addition; MC, moderate level of compost addition; HC, high level of compost addition.*

### Response of Fungal Community Composition to Compost Addition

**FIGURE 1:**
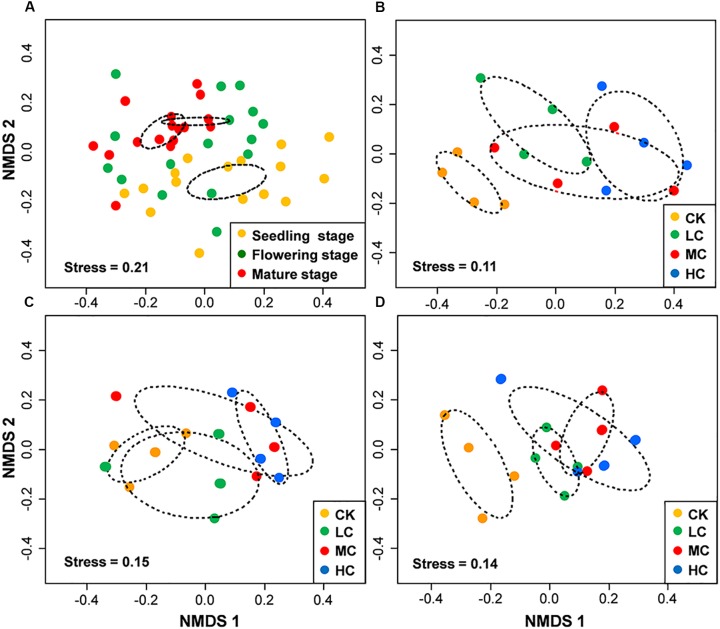
Non-metric multidimensional scaling (NMDS) of fungal community composition among growth stages **(A)**, among treatments in seedling stage **(B)**, flowering stage **(C)**, and mature stage **(D)**. Circles with dashed line in NMDS plot are 95% confidence ellipses. CK, control; LC, low level of compost addition; MC, moderate level of compost addition; HC, high level of compost addition.

PERMANOVA analysis indicated that fungal community composition was significantly influenced by compost addition (*r*^2^ = 0.14, *P* < 0.001), growth stage (*r*^2^ = 0.08, *P* < 0.001), and their interaction (*r*^2^ = 0.04, *P* = 0.013). Based on NMDS ordination, fungal communities of the flowering and mature stage overlapped, but were distinct from the seedling stage (Figure 1A). Furthermore, fungal communities in treatment CK significantly differed from treatment LC, MC, and HC in seedling and mature stage (Figure 1B,D). In flowering stage, fungal communities in treatment CK only differed from treatment HC, and overlapped with treatment LC and MC (Figure 1C). Mantel test analysis revealed that fungal community composition was significantly correlated with SOM, TN, and AP in seedling stage, correlated with SOM and AN in flowering stage, whereas no correlation was observed with any of these soil variables in mature stage (Table 2).

**Table 2:** Mantel tests of the soil fungal community with soil variables in seedling, flowering, and mature stage.

	Seedling		Flowering		Mature	
	r	P	r	P	r	P
SM	0.15	0.14	0.20	0.13	0.03	0.36
pH	−0.11	0.79	0.03	0.41	0.08	0.26
BD	0.13	0.21	−0.09	0.72	−0.11	0.73
SOM	0.26	0.04	0.40	0.006	0.01	0.43
TP	0.26	0.12	−0.13	0.81	−0.13	0.75
TN	0.31	0.005	−0.15	0.83	0.10	0.25
AP	0.43	<0.001	0.01	0.43	0.12	0.18
AN	0.03	0.38	0.23	0.03	−0.06	0.59
AK	0.19	0.06	−0.05	0.63	−0.17	0.81
C/N	0.11	0.22	0.36	0.32	0.10	0.27

*SM, soil moisture; BD, bulk density; SOM, soil organic matter; TN, total nitrogen; TP, total phosphorus; AP, available phosphorus; AN, available nitrogen; AK, available potassium; C/N, carbon nitrogen ratio.*

### Response of Dominant Fungal Taxa to Compost Addition

**FIGURE 2:**
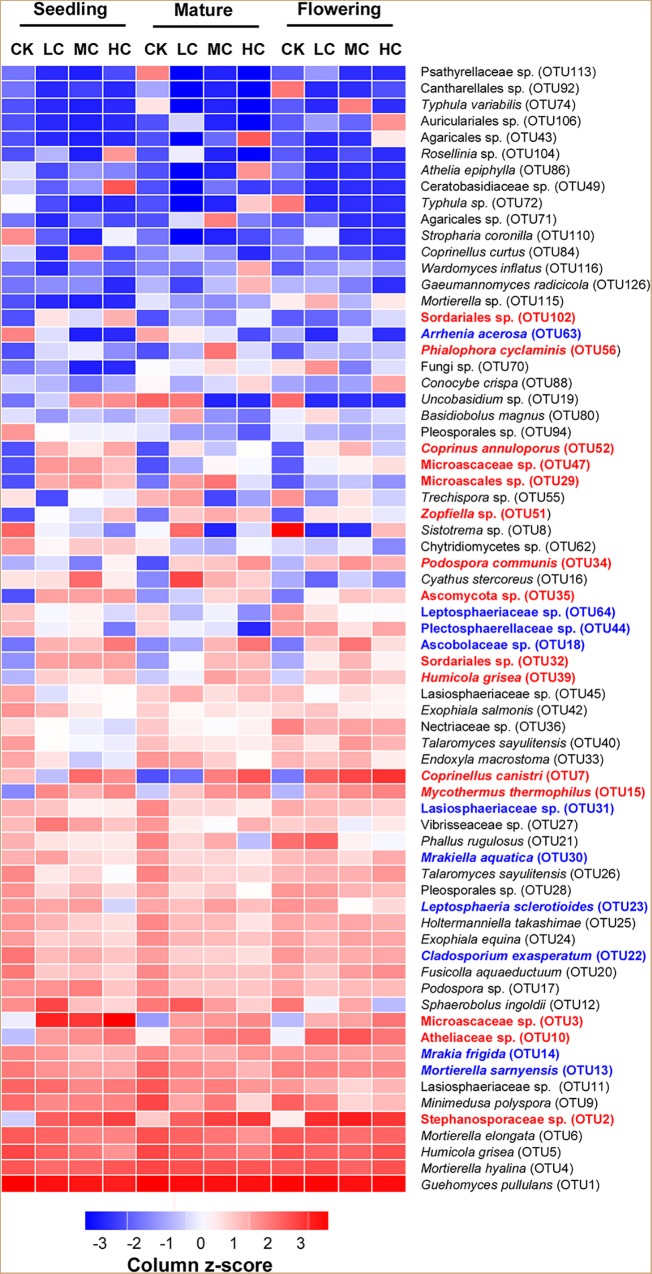
Relative abundance [log_2_ (x+0.01)] of the combined “top 20” fungal OTUs among treatments in seedling, flowering, and mature stage. Color gradients indicate the relative abundance of OTUs, with red colors indicating high abundant taxa and blue colors indicating low abundant taxa in soil. The name of OTUs in blue or red showed significantly (*P* < 0.05) lower (blue) or higher (red) relative abundances in compost amended soils (LC, MC, and HC) than in CK. See Figure 1 for the abbreviations.

Three fungal phyla including Basidiomycota, Ascomycota, and Mortierellomycota were predominant in all treatments and accounted for 97.5% of the total fungal sequences (Supplementary Figure S1). However, none of these fungal phyla was significantly affected by compost addition across the growing seasons (Supplementary Figure S1 and Supplementary Table S3), except for a slight increase observed with Ascomycota relative abundance in treatment HC compared with CK (Supplementary Figure S1). At the genus level, a total of 289 identified fungal genera (accounted for 67.15% of total fungal sequences) were obtained. Among the abundant fungal genera (relative abundance >0.5%), *Retroconis* was observed as an indicator genus for treatment CK in mature stage, while no indicator genus was found in seedling and flowering stages (Supplementary Table S4). The combined “top 20” abundant OTUs accounted for 79.60% of total fungal sequences. Among these OTUs, 15 OTUs exhibited significantly higher abundances in treatment LC, MC, and/or HC than in the control, while seven OTUs among them exhibited consistent enrichment across the growing stages (Figure 2 and Supplementary Table S5). Furthermore, 10 fungal OTUs exhibited significantly lower abundance in compost amended soils compared with the control in either seedling and/or flowering stage (Figure 2 and Supplementary Table S5), but did not occur in the mature stage.

### Predicted Fungal Function

Soil fungal community was assessed in terms of fungal guilds, and 45.5% of fungal OTUs were assigned to a fungal guild. Kruskal–Wallis analysis revealed that the abundance of pathotroph was significantly influenced by compost addition in seedling stage (Supplementary Table S6). In seedling stage, treatment HC showed a decreased pathotroph abundance of 85.3% as compared with CK (Supplementary Figure S2 and Supplementary Table S6). However, the abundance of saprotroph and symbiotroph were unaffected by compost addition across the growing season (Supplementary Figure S2).

### Network Analysis of Soil Fungal Communities

**FIGURE 3:**
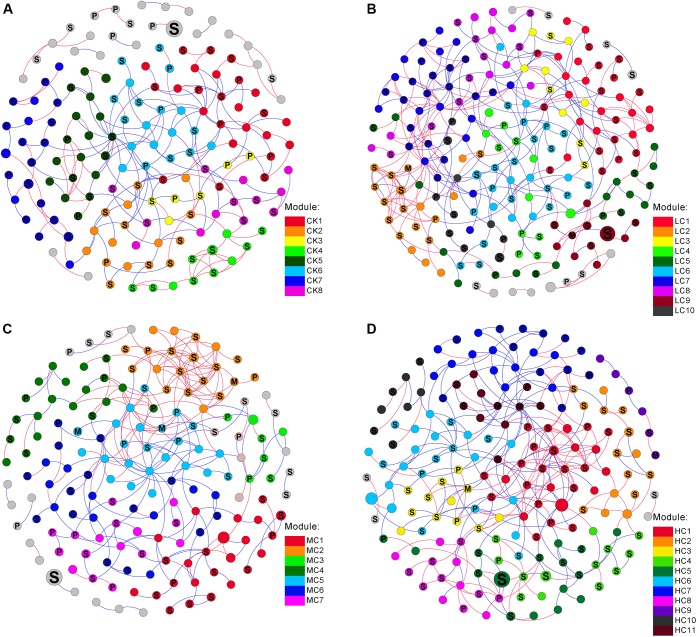
Networks of fungal communities in control **(A)**, low level of compost **(B)**, moderate level of compost **(C)**, and high level of compost **(D)** amended soils. The size of each node is proportional to the relative abundance. Modules larger than five nodes are labeled with different colors in the respective network. The nodes were labeled as M (symbiotroph), P (pathotroph), and S (saprotroph) as predicted by FUNGuild. The red and blue lines indicate positive and negative connections between the nodes, respectively.

The networks of soil fungal community among treatments were analyzed (Figure 3) and main topological properties are shown in Table 3. The similarity threshold was adjusted to 0.75 in all networks to allow comparison. Connectivity was well-fitted by the power-law (*R*^2^ values ranged from 0.795 to 0.897), indicating scale-free properties of the networks. All networks were modular, as their modularity values were higher than those of their corresponding randomized networks (Table 3). The composition of CK network differed greatly from LC, MC, and HC network, with 111 (71.6.9%), 83 (53.5%), and 86 nodes (55.5%) shared, respectively. The LC, MC, and HC networks were more complex and better connected than CK network (Figure 3), which was confirmed by the topological properties (Table 3). In addition, the ratio of positive links to negative links was higher in LC, MC, and HC networks than CK (Figure 3 and Table 3).

**Table 3:** Major topological properties of the empirical molecular ecological networks (MENs) of soil fungal communities in CK, LC, MC, and HC treatment and their associated random MENs.

		CK	LC	MC	HC
Empirical networks	No. of original OTUs	207	233	220	213
	Total nodes	155	200	160	174
	Total links	220	330	264	302
	R^2^ of power-law	0.80	0.88	0.90	0.84
	Average degree	2.84	3.3	3.3	3.47
	Average clustering coefficient	0.09	0.12	0.14	0.11
	Average path distance	5.23	5.07	5.16	4.85
	Modularity	0.70	0.66	0.68	0.65
	Connectedness	0.75	0.88	0.81	0.89
	Positive links/negative links	0.71	0.80	1.08	0.79
Random networks	Average path distance	4.66 ± 0.13	4.22 ± 0.08	3.97 ± 0.08	4.01 ± 0.07
	Average cluster coefficient	0.02 ± 0.01	0.02 ± 0.01	0.03 ± 0.01	0.02 ± 0.01
	Modularity	0.62 ± 0.01	0.56 ± 0.01	0.54 ± 0.010	0.53 ± 0.01

Trophic status was used to characterize the co-occurence network patterns. We observed that the proportion of symbiotroph gradually increased with increasing compost rate, while the pathotroph exhibited the opposite trend (Figure 3). To assess the potential interactions among different trophic modes, the positive and negative links of each guild pair was counted. The proportion of positive links among saprotroph was greater in MC (65.7%) than those in CK (47.8%), LC (48.5%), and HC (47.7%) networks (Figure 3). In addition, saprotroph tended to be more positively linked with symbiotroph in MC network in comparison with the other networks (Figure 3).

**FIGURE 4:**
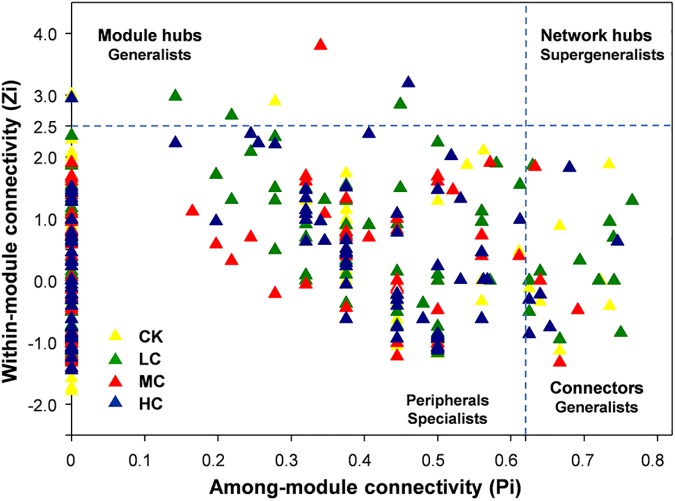
Zi-Pi plot showing the distribution of soil fungal OTUs based on their topological roles. Each triangle represents an OTU in CK, LC, MC, or HC network. See Figure 1 for the abbreviations.

From the plot of Zi (a value measuring within-module connectivity) and Pi (a value measuring among-module connectivity), the different roles of each node in the network were identified (Figure 4). The vast majority of nodes were categorized as “peripherals” in all networks (Figure 4), others were categorized as “module hubs” and “connectors.” Notably, more “connectors” were observed in CK (11) and LC (15) networks than in MC (4) and HC (6) networks (Figure 4). The phylogenetic classification of each module hub and connector is shown in Supplementary Table S7.

## Discussion

A number of recently published studies indicate that ecosystem functioning is positively related to fungal diversity, and the loss of fungal diversity can affect ecosystem functions, including enzyme activities and leaf litter decomposition (Hoppe et al., 2016; Bani et al., 2018). Therefore, insight into fungal microbial diversity is critical for the understanding of agroecosystem functioning (Njeru et al., 2015). In this study, our results indicated that the fungal OTU richness and diversity were unaffected by compost addition across the growing seasons, which was contrary to our hypothesis. Although a positive effect of organic amendment on fungal diversity has been consistently reported (Hartmann et al., 2015; Song et al., 2015; Hu et al., 2017), non-effect or negative effect of organic amendment has also been observed (Bonanomi et al., 2016; Sun et al., 2016; Ding et al., 2017; Zhang Y.T. et al., 2018). Black soil is characterized by high organic matter content (Giguere et al., 2017), and the SOM content ranged from 22.01 (CK) to 48.20 g/kg (HC) in the present study. Therefore, the high SOM content may possibly weaken the effect of compost addition on fungal diversity. Alternatively, the non-effect of compost addition might have been due to the relatively short experiment duration (Hartmann et al., 2015; Song et al., 2015; Hu et al., 2017).

Unlike soil fungal richness and diversity, community structure responded to compost addition across the growing season. This observation corroborated the results of Xue et al. (2018) and Hartmann et al. (2015) who reported that organic amendment application significantly influenced soil fungal communities in agroecosystems. It has been suggested that variation in fungal communities after organic amendment application is possible due to the shift in soil nutrient availability (Marschner et al., 2003; Sun et al., 2016). As revealed by the Mantel test, the effect of compost addition on fungal community composition was mainly mediated through soil pH and TP content. In addition, the compost addition could introduce exogenous microorganisms into native soil (Sun et al., 2016). Although it was argued that the native microbial community will out compete that from compost (Saison et al., 2006), some studies have shown that exogenous microbes introduced to soil from manure or compost had an observed influence on soil microbial communities (Unc and Goss, 2004; Sun et al., 2016). It has been reported that *Mycothermus thermophilus* is one of the most important thermophilic fungi during the cow manure composting process (Wang et al., 2018). In this study, *M. thermophilus* was not abundant (<10 reads) in the control soil, but observed to be significantly enriched in compost amended soils (>120 reads). Therefore, the shifts in fungal community composition caused by compost addition were possibly not only due to the nutrient change, but also to the existing fungi present in the compost.

Our results indicated that abundant fungal OTUs exhibited contrasting response to compost addition. For instance, OTU 39, which assigned to genus *Humicola*, was observed to increase with increasing compost rate. Members of this genus were reported to have the potential for biological control of plant diseases, and have been found to be the predominant genus in straw or manure compost amended soils (Lang et al., 2012; Banerjee et al., 2016). OTU 34, member in *Podospora*, was found to be significantly enriched in compost amended soils in the present study. This observation also agrees with the results found by Hartmann et al. (2015) and Ding et al. (2017). *Podospora* has generally been reported to be beneficial, due to the production of antifungal agents by taxa in this genus (Che et al., 2002). The application of compost is also known to suppress soil-borne plant pathogens, due to its chemical and biological characteristics (Pane et al., 2013). Interestingly, some potential plant pathogens, such as *Cladosporium exasperatum* and *Leptosphaeria sclerotioides* were substantially decreased by compost addition. These results were further confirmed by the results of predicted functional groups of fungi characterized by FUNGuild. Taken as a whole, the results suggest that the modified community composition of soil fungi due to compost addition is beneficial for soil health maintenance.

In addition to changes in soil fungal community composition, compost amendment also influenced the fungal co-occurrence network patterns. Our result indicated that the LC, MC, and HC networks exhibited greater complexity than the control, reflected by the greater number of nodes, links and connectivity. The enhanced network complexity in compost amended soils is unlikely to be a consequence of tighter fungal hyphal connections, because these are probably disrupted by the tillage practice applied in present study. Therefore, the complexed fungal network is more likely due to a combination of increased and more balanced fertility, as well as the synergetic interactions promoted by the biotic community added to the soil together with the compost. It has been proposed that a highly connected network provides more functional redundancy (Mougi and Kondoh, 2012). Therefore, this suggests that the complex fungal network in compost amended soils would lead to greater community stability and thus provide stronger resistance to disturbance (Scheffer et al., 2012). Additionally, Morrien et al. (2017) suggested that a more connected network may increase the efficient utilization of carbon. In our study, the exchange of nutrients among different soil fungal species could have possibly been enhanced by the compost amendment. The CK network had many negative links, suggesting that these fungal species could be competing for resources (Fuhrman, 2009). Whereas, the compost amendment may have alleviated the competition and provided more heterogeneous niches for soil fungi (Banerjee et al., 2016), causing the number of negative links to substantially decrease in the compost amended soils.

Furthermore, we used trophic status to illustrate the fungal co-occurrence network patterns. Most of the nodes predicted as symbiotroph in the present study were identified as arbuscular mycorrhizal fungi (AMF). We observed that the proportion of symbiotroph involved in the co-occurrence networks gradually increased along with compost rate, possibly due to enhancement of AM fungal biomass by compost addition (Yang et al., 2018). Interestingly, the MC network harbored more positive links among saprotroph-saprotroph and saprotroph-symbiotroph than other networks. This finding suggests that the moderate but not high level of compost addition enhance the synergistic interactions among saprotroph-saprotroph and saprotroph-symbiotroph.

In the present study, module hubs, and connectors were identified in all networks. These generalists bridged different nodes within their own modules and/or among different modules, whereas specialists linked to only a few nodes (Deng et al., 2012; Ling et al., 2016). Therefore, these generalists may play key roles in promoting exchanges of nutrients and metabolites among different fungal species in networks (Olesen et al., 2007). Furthermore, roles of some nodes shifted in the four networks. For instance, OTU101, OTU 136, and OTU 255 were observed to be generalists in HC network, but specialists in CK, LC, MC, and HC networks, suggesting that compost amendment may have changed the ecological roles of key soil fungi.

## Conclusion

In conclusion, our study revealed that soil fungal alpha-diversity indices were resistant to compost addition in all the growth stages. However, soil fungal community was consistently affected by compost addition across the growing season. The shift in soil fungal community was also reflected in the alteration of abundant OTUs, which exhibited various response to compost addition. As predicted by FUNGuild, the abundance of pathotroph was greatly decreased by the 45 Mg ha^−1^ compost addition. Notably, the fungal networks in compost amended soils were more complex and harbored more positive links than the control. Overall, our findings show that 1-year compost addition maintains the soil fungal alpha-diversity but alters the fungal community composition and network patterns in black soil of Northeast China.

## Author Contributions

WY and SG planned and designed the research. WY, YG, and CZ carried out the research. TW, DS, and WS conducted the fieldwork. WY, XJ, and SG wrote the manuscript.

## Conflict of Interest Statement

The authors declare that the research was conducted in the absence of any commercial or financial relationships that could be construed as a potential conflict of interest.
