# Supplementary figures and images for: Response of Fungal Communities and Co-occurrence Network Patterns to Compost Amendment in Black Soil of Northeast China
Source: Front Microbiol. 2019 Jul 9;10:1562. doi: 10.3389/fmicb.2019.01562 (PMC6629936; doi:10.3389/fmicb.2019.01562)

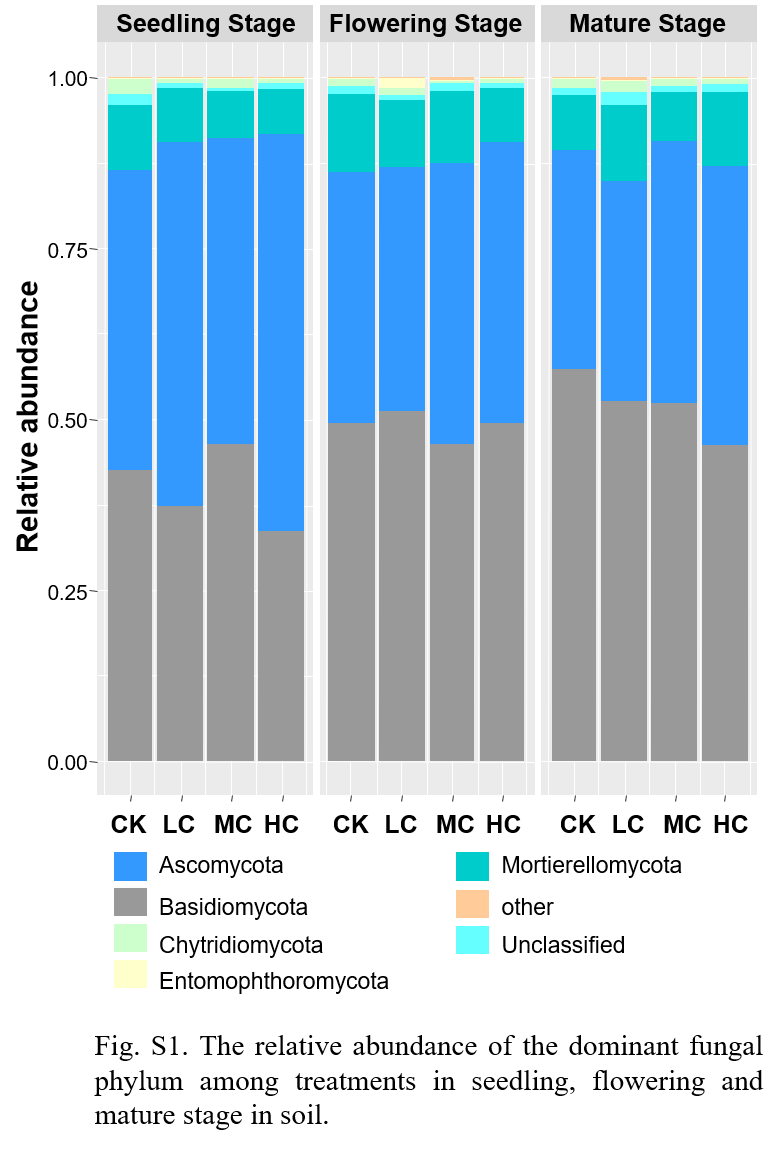

Supplement: Supplementary file 1 [file Image_1.PNG]

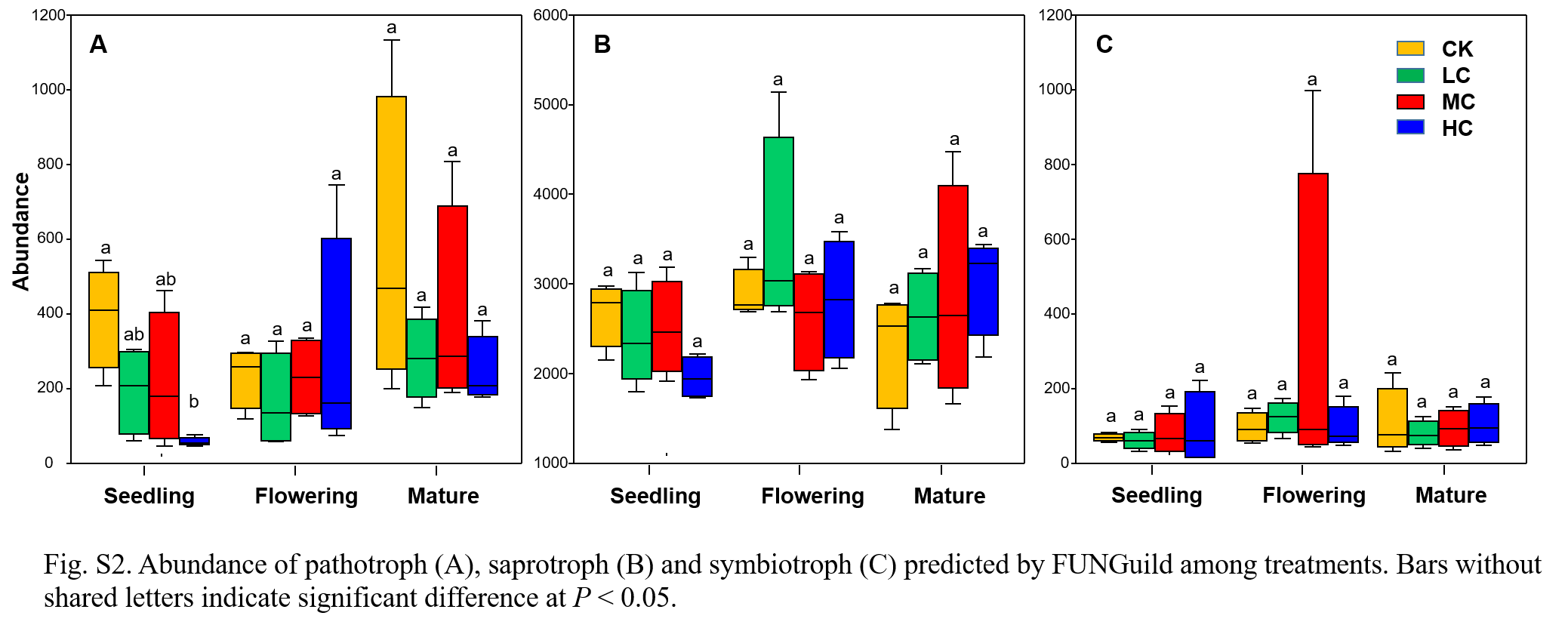

Supplement: Supplementary file 2 [file Image_3.PNG]
