# Supplementary material for: Response of Fungal Communities and Co-occurrence Network Patterns to Compost Amendment in Black Soil of Northeast China
Source: Front Microbiol. 2019 Jul 9;10:1562. doi: 10.3389/fmicb.2019.01562 (PMC6629936; doi:10.3389/fmicb.2019.01562)
Supplement: Supplementary file 3 [file Table_1.docx]

**Supplementary Table S1**

**PCR conditions:**

The amplification was carried out in a final 25 μL reaction solution including 2.5 μL of 10 × buffer, 1.5 mM of MgCl_2_, 200 μM of each dNTP, 0.75 μM of each primer, 0.75 U PrimeSTAR HS DNA Polymerase (Takara, Japan), and 1μL of template DNA. The ITS was amplified with the following conditions: denaturation at 94 °C for 5 min, 30 cycles of denaturation at 94 °C for 30 s, annealing at 56 °C for 30 s, and extension at 68 °C for 45 s, followed by a final extension at 72 °C for 10 min. All PCR products were purified using an agarose gel DNA purification kit (TaKaRa, Japan) and quantified using Nanodrop 2000 (Thermoscientific, USA). Only PCR products with concentration > 10 ng/μL and OD 260 / OD 280≈1.8 were used, others were discarded and re-amplified to ensure the Miseq sequencing quality. The final PCR products from all samples were mixed at equimolar concentrations and then subjected to Illumina Miseq platform at Environmental Genome Platform of Chengdu Institute of Biology, Chinese Academy of Sciences.
