# Supplementary material for: Response of Fungal Communities and Co-occurrence Network Patterns to Compost Amendment in Black Soil of Northeast China
Source: Front Microbiol. 2019 Jul 9;10:1562. doi: 10.3389/fmicb.2019.01562 (PMC6629936; doi:10.3389/fmicb.2019.01562)
Supplement: Supplementary file 4 [file Table_2.DOCX]

| **TABLE S2**  One way ANOVA examining the effect of compost addition (C) on the OTU richness (*S*), Shannon diversity (*H*), Simpson diversity (D) and Pielou evenness (*J*) index of soil fungi. | | | | | | | | |
| --- | --- | --- | --- | --- | --- | --- | --- | --- |
|  | Seedling | |  | Flowering | |  | Mature | |
| Variables | *F* | *P* |  | *F* | *P* |  | *F* | *P* |
| *S* | 2.51 | 0.11 |  | 0.16 | 0.92 |  | 0.94 | 0.45 |
| *H* | 2.48 | 0.11 |  | 0.49 | 0.70 |  | 1.60 | 0.24 |
| *J* | 1.87 | 0.19 |  | 0.14 | 0.93 |  | 0.83 | 0.50 |
| *D* | 0.72 | 0.56 |  | 0.51 | 0.68 |  | 1.29 | 0.32 |
