# Supplementary material for: Response of Fungal Communities and Co-occurrence Network Patterns to Compost Amendment in Black Soil of Northeast China
Source: Front Microbiol. 2019 Jul 9;10:1562. doi: 10.3389/fmicb.2019.01562 (PMC6629936; doi:10.3389/fmicb.2019.01562)
Supplement: Supplementary file 5 [file Table_3.DOCX]

| **TABLE S3**  One way ANOVA examining the effects of compost addition (C) on the abundant phyla of soil fungi in seedling, flowering and mature stage. | | | | | | | | |
| --- | --- | --- | --- | --- | --- | --- | --- | --- |
|  | Seedling | |  | Flowering | |  | Mature | |
| Phylum | *F* | *P* |  | *F* | *P* |  | *F* | *P* |
| Ascomycota | 2.55 | 0.10 |  | 0.25 | 0.86 |  | 1.77 | 0.21 |
| Basidiomycota | 0.89 | 0.47 |  | 0.96 | 0.44 |  | 0.47 | 0.71 |
| Chytridiomycota | 0.84 | 0.50 |  | 0.98 | 0.43 |  | 1.02 | 0.42 |
| Entomophthoromycota | 0.35 | 0.79 |  | 1.56 | 0.25 |  | 1.02 | 0.42 |
| Glomeromycota | 0.53 | 0.67 |  | 1.04 | 0.41 |  | 1.65 | 0.23 |
| Mortierellomycota | 0.27 | 0.84 |  | 0.90 | 0.47 |  | 1.17 | 0.36 |
