# Supplementary material for: Response of Fungal Communities and Co-occurrence Network Patterns to Compost Amendment in Black Soil of Northeast China
Source: Front Microbiol. 2019 Jul 9;10:1562. doi: 10.3389/fmicb.2019.01562 (PMC6629936; doi:10.3389/fmicb.2019.01562)
Supplement: Supplementary file 6 [file Table_4.DOCX]

| Table S4 Indicator genera of soil fungi for different treatments in seedling, flowering and mature stage. | | | | | | | | | |
| --- | --- | --- | --- | --- | --- | --- | --- | --- | --- |
|  | Seedling stage | | | Flowering stage | | | Mature stage | | |
| Genus | Treatment | Indval value | *P* value | Treatment | Indval value | *P* value | Treatment | Indval value | *P* value |
| *Chaetomidium* | HC | 0.31 | 0.72 | CK | 0.37 | 0.74 | CK | 0.39 | 0.06 |
| *Cladophialophora* | MC | 0.48 | 0.59 | MC | 0.42 | 0.24 | MC | 0.33 | 0.67 |
| *Cladosporium* | HC | 0.34 | 0.38 | MC | 0.33 | 0.59 | LC | 0.33 | 0.29 |
| *Coprinellus* | HC | 0.57 | 0.32 | CK | 0.45 | 0.5 | HC | 0.51 | 0.36 |
| *Coprinopsis* | HC | 0.46 | 0.78 | MC | 0.45 | 0.81 | LC | 0.42 | 0.21 |
| *Coprinus* | LC | 0.55 | 0.18 | CK | 0.52 | 0.47 | LC | 0.48 | 0.34 |
| *Cyathus* | HC | 0.65 | 0.67 | LC | 0.69 | 0.64 | MC | 0.51 | 0.19 |
| *Endoxyla* | CK | 0.6 | 0.08 | LC | 0.33 | 0.64 | MC | 0.37 | 0.22 |
| *Exophiala* | HC | 0.37 | 0.35 | MC | 0.29 | 0.78 | MC | 0.3 | 0.77 |
| *Fusicolla* | MC | 0.39 | 0.83 | CK | 0.33 | 0.85 | CK | 0.38 | 0.48 |
| *Guehomyces* | HC | 0.37 | 0.24 | HC | 0.3 | 0.87 | CK | 0.38 | 0.17 |
| *Holtermanniella* | MC | 0.32 | 0.59 | CK | 0.3 | 0.95 | CK | 0.4 | 0.14 |
| *Humicola* | HC | 0.28 | 0.93 | CK | 0.33 | 0.93 | CK | 0.37 | 0.28 |
| *Leptosphaeria* | HC | 0.57 | 0.49 | LC | 0.34 | 0.85 | LC | 0.33 | 0.96 |
| *Minimedusa* | LC | 0.64 | 0.09 | MC | 0.66 | 0.17 | MC | 0.41 | 0.54 |
| *Mortierella* | LC | 0.33 | 0.38 | HC | 0.27 | 0.96 | CK | 0.42 | 0.2 |
| *Mrakia* | MC | 0.3 | 0.74 | HC | 0.37 | 0.2 | CK | 0.32 | 0.57 |
| *Mrakiella* | MC | 0.34 | 0.4 | HC | 0.41 | 0.19 | CK | 0.52 | 0.08 |
| *Mycothermus* | LC | 0.49 | 0.15 | MC | 0.32 | 0.74 | LC | 0.37 | 0.62 |
| *Phallus* | HC | 0.41 | 0.44 | HC | 0.5 | 0.64 | CK | 0.5 | 0.73 |
| *Phialophora* | CK | 0.4 | 0.95 | LC | 0.94 | 0.17 | CK | 0.74 | 0.08 |
| *Podospora* | HC | 0.5 | 0.32 | MC | 0.36 | 0.67 | LC | 0.31 | 0.74 |
| *Retroconis* | LC | 0.41 | 0.55 | LC | 0.39 | 0.39 | CK | 0.4 | 0.04 |
| *Sistotrema* | MC | 0.93 | 0.14 | CK | 0.24 | 1 | HC | 0.74 | 0.09 |
| *Sphaerobolus* | MC | 0.34 | 0.8 | MC | 0.43 | 0.75 | HC | 0.46 | 0.88 |
| *Talaromyces* | CK | 0.31 | 0.99 | CK | 0.31 | 0.81 | CK | 0.34 | 0.78 |
| *Trichoderma* | HC | 0.34 | 0.72 | HC | 0.34 | 0.61 | LC | 0.31 | 0.68 |
| *Uncobasidium* | HC | 0.4 | 0.35 | MC | 0.28 | 0.41 | MC | 0.25 | 1 |
| *Zopfiella* | LC | 0.41 | 0.45 | CK | 0.54 | 0.19 | CK | 0.46 | 0.3 |
