# Supplementary material for: Response of Fungal Communities and Co-occurrence Network Patterns to Compost Amendment in Black Soil of Northeast China
Source: Front Microbiol. 2019 Jul 9;10:1562. doi: 10.3389/fmicb.2019.01562 (PMC6629936; doi:10.3389/fmicb.2019.01562)
Supplement: Supplementary file 7 [file Table_5.DOCX]

| **TABLE S5**  Kruskal-Willis examining the effects of compost addition (C) on the abundant OTUs of fungi in seedling, flowering and mature stage. | | | | | | | | | |
| --- | --- | --- | --- | --- | --- | --- | --- | --- | --- |
| Variables | Taxonomic | Seedling | |  | Flowering | |  | Mature | |
|  |  | *X*^2^ | *P* |  | *X*^2^ | *P* |  | ***X*^2^** | *P* |
| OTU1 | *Guehomyces pullulans* | 5.67 | 0.129 |  | 2.49 | 0.477 |  | 2.8 | 0.423 |
| OTU2 | *Stephanosporaceae* sp. | 10.34 | 0.016 |  | 9.39 | 0.025 |  | 8.89 | 0.031 |
| OTU3 | *Microascaceae* sp. | 12.26 | 0.007 |  | 9.05 | 0.029 |  | 10.51 | 0.016 |
| OTU4 | *Mortierella hyalina* | 0.15 | 0.985 |  | 1.08 | 0.782 |  | 1.81 | 0.613 |
| OTU5 | *Humicola grisea* | 5.38 | 0.146 |  | 0.48 | 0.477 |  | 1.48 | 0.687 |
| OTU6 | *Mortierella elongata* | 3.26 | 0.353 |  | 7.3 | 0.063 |  | 3.51 | 0.32 |
| OTU7 | *Coprinellus canistri* | 3.99 | 0.263 |  | 7.86 | 0.049 |  | 3.13 | 0.371 |
| OTU8 | *Sistotrema* sp. | 3.88 | 0.275 |  | 1.95 | 0.583 |  | 7.34 | 0.062 |
| OTU9 | *Minimedusa polyspora* | 0.68 | 0.877 |  | 5.36 | 0.147 |  | 6 | 0.112 |
| OTU10 | *Atheliaceae* sp. | 10.81 | 0.013 |  | 9.23 | 0.026 |  | 8.64 | 0.035 |
| OTU11 | *Lasiosphaeriaceae* sp. | 2.4 | 0.493 |  | 1.39 | 0.708 |  | 5.08 | 0.166 |
| OTU12 | *Sphaerobolus ingoldii* | 0.66 | 0.884 |  | 1.63 | 0.653 |  | 2.35 | 0.503 |
| OTU13 | *Mortierella sarnyensis* | 4.16 | 0.248 |  | 11.32 | 0.01 |  | 3.01 | 0.39 |
| OTU14 | *Mrakia frigida* | 8.6 | 0.035 |  | 0.87 | 0.833 |  | 0.73 | 0.866 |
| OTU15 | *Mycothermus thermophilus* | 9.56 | 0.023 |  | 11.42 | 0.01 |  | 9.26 | 0.026 |
| OTU16 | *Cyathus stercoreus* | 1.37 | 0.713 |  | 2.93 | 0.403 |  | 2.52 | 0.471 |
| OTU17 | *Podospora* sp. | 2.79 | 0.425 |  | 2.73 | 0.435 |  | 0.28 | 0.964 |
| OTU18 | *Ascobolaceae* sp. | 9.65 | 0.022 |  | 9.14 | 0.028 |  | 6.61 | 0.085 |
| OTU19 | *Uncobasidium* sp. | 1.14 | 0.767 |  | 1.19 | 0.756 |  | 3 | 0.392 |
| OTU20 | *Fusicolla aquaeductuum* | 3.13 | 0.372 |  | 0.43 | 0.699 |  | 3.86 | 0.277 |
| OTU21 | *Phallus rugulosus* | 0.91 | 0.822 |  | 4.2 | 0.24 |  | 7.13 | 0.068 |
| OTU22 | *Cladosporium exasperatum* | 10.63 | 0.014 |  | 1.26 | 0.74 |  | 0.49 | 0.92 |
| OTU23 | *Leptosphaeria sclerotioides* | 6.64 | 0.084 |  | 0.93 | 0.82 |  | 8.55 | 0.036 |
| OTU24 | *Exophiala equina* | 4.23 | 0.238 |  | 2.4 | 0.495 |  | 1.43 | 0.698 |
| OTU25 | *Holtermanniella takashimae* | 7.22 | 0.065 |  | 4.37 | 0.224 |  | 0.24 | 0.97 |
| OTU26 | *Talaromyces sayulitensis* | 6.1 | 0.107 |  | 0.97 | 0.807 |  | 3.6 | 0.309 |
| OTU27 | *Vibrisseaceae* sp. | 0.22 | 0.947 |  | 5.29 | 0.152 |  | 4.43 | 0.219 |
| OTU28 | *Pleosporales* sp. | 6.18 | 0.103 |  | 4.72 | 0.193 |  | 1.03 | 0.793 |
| OTU29 | *Microascales* sp. | 9.15 | 0.027 |  | 7.32 | 0.063 |  | 7.29 | 0.063 |
| OTU30 | *Mrakiella aquatica* | 6.34 | 0.096 |  | 7.79 | 0.05 |  | 3.7 | 0.296 |
| OTU31 | *Lasiosphaeriaceae* sp. | 8.34 | 0.039 |  | 4.7 | 0.195 |  | 2.54 | 0.469 |
| OTU32 | *Sordariales* sp. | 8.18 | 0.042 |  | 8.03 | 0.045 |  | 7.27 | 0.064 |
| OTU33 | *Endoxyla macrostoma* | 6.56 | 0.087 |  | 2.16 | 0.54 |  | 1.81 | 0.613 |
| OTU34 | *Podospora communis* | 3.03 | 0.387 |  | 8.02 | 0.046 |  | 10.21 | 0.017 |
| OTU35 | *Ascomycota* sp. | 9.02 | 0.029 |  | 11.72 | 0.008 |  | 9.12 | 0.027 |
| OTU36 | *Nectriaceae* sp. | 4.13 | 0.248 |  | 1.27 | 0.738 |  | 0.42 | 0.936 |
| OTU39 | *Humicola grisea* | 8.81 | 0.032 |  | 10.72 | 0.013 |  | 8.11 | 0.044 |
| OTU40 | *Talaromyces sayulitensis* | 1.21 | 0.75 |  | 0.27 | 0.966 |  | 3.16 | 0.368 |
| OTU42 | *Exophiala salmonis* | 6.04 | 0.109 |  | 0.51 | 0.917 |  | 0.95 | 0.814 |
| OTU43 | *Agaricales* sp. | 0 | 1 |  | 2.15 | 0.542 |  | 3 | 0.392 |
| OTU44 | *Plectosphaerellaceae* sp. | 6.27 | 0.099 |  | 9.92 | 0.019 |  | 3.2 | 0.361 |
| OTU45 | *Lasiosphaeriaceae* sp. | 5.12 | 0.163 |  | 0.55 | 0.908 |  | 0.99 | 0.803 |
| OTU47 | *Microascaceae* sp. | 7.6 | 0.055 |  | 10.92 | 0.012 |  | 9.12 | 0.028 |
| OTU49 | *Ceratobasidiaceae* sp. | 3.16 | 0.367 |  | 2.15 | 0.542 |  | 0 | 1 |
| OTU51 | *Zopfiella* sp. | 10.18 | 0.017 |  | 7.54 | 0.057 |  | 6.67 | 0.083 |
| OTU52 | *Coprinus annuloporus* | 9.32 | 0.025 |  | 7.96 | 0.047 |  | 10.61 | 0.014 |
| OTU55 | *Trechispora* sp. | 4.56 | 0.207 |  | 2.82 | 0.42 |  | 2.88 | 0.411 |
| OTU56 | *Phialophora cyclaminis* | 6.96 | 0.073 |  | 10.67 | 0.014 |  | 7.67 | 0.053 |
| OTU62 | *Chytridiomycetes* sp. | 2.09 | 0.553 |  | 5.28 | 0.152 |  | 1.44 | 0.696 |
| OTU63 | *Arrhenia acerosa* | 10.82 | 0.013 |  | 0.32 | 0.956 |  | 10.77 | 0.013 |
| OTU64 | *Leptosphaeriaceae* sp. | 8.64 | 0.034 |  | 10.25 | 0.017 |  | 0.45 | 0.93 |
| OTU70 | *Fungi* sp. | 2.15 | 0.542 |  | 0.42 | 0.935 |  | 0.77 | 0.856 |
| OTU71 | *Agaricales* sp. | 1.18 | 0.758 |  | 3.15 | 0.37 |  | 3 | 0.392 |
| OTU72 | *Typhula* sp. | 4.6 | 0.203 |  | 3 | 0.392 |  | 3 | 0.392 |
| OTU74 | *Typhula variabilis* | 0 | 1 |  | 3 | 0.392 |  | 3 | 0.392 |
| OTU80 | *Basidiobolus magnus* | 0.53 | 0.912 |  | 3.2 | 0.362 |  | 0.23 | 0.973 |
| OTU84 | *Coprinellus curtus* | 4.81 | 0.186 |  | 1.47 | 0.69 |  | 1.18 | 0.758 |
| OTU86 | *Athelia epiphylla* | 1.41 | 0.704 |  | 3 | 0.392 |  | 3 | 0.392 |
| OTU88 | *Conocybe cri*sp.*a* | 2.62 | 0.453 |  | 1.99 | 0.574 |  | 1.53 | 0.675 |
| OTU92 | *Cantharellales* sp. | 2.14 | 0.543 |  | 6.4 | 0.094 |  | 2.15 | 0.542 |
| OTU94 | *Pleosporales* sp. | 4.23 | 0.237 |  | 1.3 | 0.729 |  | 0.6 | 0.897 |
| OTU102 | *Sordariales* sp. | 8.69 | 0.034 |  | 9.07 | 0.028 |  | 6.81 | 0.078 |
| OTU104 | *Rosellinia* sp. | 5.03 | 0.17 |  | 3 | 0.392 |  | 3 | 0.392 |
| OTU106 | *Auriculariales* sp. | 0 | 1 |  | 6.4 | 0.094 |  | 2.62 | 0.453 |
| OTU110 | *Stropharia coronilla* | 2.58 | 0.461 |  | 2.15 | 0.542 |  | 4.39 | 0.222 |
| OTU113 | *Psathyrellaceae* sp. | 2.14 | 0.543 |  | 6.4 | 0.094 |  | 3 | 0.392 |
| OTU115 | *Mortierella* sp. | 0 | 1 |  | 0.69 | 0.877 |  | 0.36 | 0.948 |
| OTU116 | *Wardomyces inflatus* | 3.13 | 0.373 |  | 3.2 | 0.362 |  | 4.84 | 0.184 |
