# Supplementary material for: Response of Fungal Communities and Co-occurrence Network Patterns to Compost Amendment in Black Soil of Northeast China
Source: Front Microbiol. 2019 Jul 9;10:1562. doi: 10.3389/fmicb.2019.01562 (PMC6629936; doi:10.3389/fmicb.2019.01562)
Supplement: Supplementary file 8 [file Table_6.DOCX]

| **Table S6**  One-way ANOVA examining the effects of compost addition on the abundance of fungal guild in seedling, flowering and mature stage. | | | | | | | | |
| --- | --- | --- | --- | --- | --- | --- | --- | --- |
|  | Seedling | |  | Flowering | |  | Mature | |
|  | *F* | *P* |  | *F* | *P* |  | *F* | *P* |
| pathotroph | 4.51 | 0.02 |  | 0.30 | 0.82 |  | 1.29 | 0.32 |
| saprotroph | 1.97 | 0.17 |  | 1.03 | 0.41 |  | 0.63 | 0.61 |
| symbiotroph | 0.18 | 0.91 |  | 0.75 | 0.54 |  | 0.17 | 0.92 |
